# ReNU Syndrome and the RNU4-2 Mutation: A Missed Cause of Childhood Neurodevelopmental Delay

**DOI:** 10.34763/jmotherandchild.20263001.d-26-00014

**Published:** 2026-05-31

**Authors:** Jawairya Muhammad Hussain, Laiba Ilyas, Areeba Amir, Muhammad Azlan

**Affiliations:** Karachi Medical and Dental College, Karachi, Pakistan; Jinnah Sindh Medical University, Karachi, Pakistan; CMH Lahore Medical College, Lahore, Pakistan

**Keywords:** ReNU Syndrome, RNU4-2 Mutation, U4 snRNA Disorder, Paediatric Genetic Screening, Neurodevelopmental Disorder

## Abstract

ReNU syndrome is a rare but increasingly recognized syndromic neurodevelopmental disorder caused by heterozygous de novo variants in the non-coding gene RNU4-2, which encodes the U4 small nuclear RNA (snRNA), a critical component of the major spliceosome. Recurrent pathogenic variants cluster within an 18-base-pair critical region of RNU4-2 and disrupt normal spliceosome function by altering 5′ splice-site usage. In large cohorts, most affected individuals harbor a recurrent single-base insertion (n.64_65insT), accounting for the majority of pathogenic variants, and RNU4-2 variants may explain ~0.4% of undiagnosed neurodevelopmental disorders. Clinically, ReNU syndrome is characterized by global developmental delay, intellectual disability, hypotonia, delayed motor milestones, limited speech, feeding difficulties, distinctive facial features, and structural brain anomalies. Given the emerging understanding of its prevalence and severe phenotype, we highlight the urgent need to incorporate RNU4-2 analysis into diagnostic genomic testing for pediatric neurodevelopmental disorders. Increasing awareness and equitable access to comprehensive genetic testing will improve diagnosis and counseling for affected families worldwide.

ReNU syndrome, a rare neurodevelopmental disorder (NDD), is estimated to contribute to approximately 0.4–0.6% of unresolved NDD cases, affecting primarily children, making it one of the more frequent monogenic causes identified in this population [1]. Hypotonia, global developmental delay, significant intellectual impairment with limited vocabulary, inability to walk, eating issues, seizures, short stature, and characteristic dysmorphic facial traits are its hallmarks. A thin corpus callosum, ventriculomegaly, and white matter atrophy are among the common brain abnormalities [1]. Although once thought to be rare, RNU4-2-related disorders may be more common than assumed. However, it remains underdiagnosed due to limited assessment of non-coding RNA genes in standard diagnostic pipelines and overlap with other NDD. Late diagnosis can result in more complications and limited access to therapy and genetic counseling. This paper highlights the urgent need to include RNU4-2 in pediatric genetic screening, as variants in this non-coding gene cause ReNU syndrome. We emphasize diagnostic gaps and call for greater awareness and equitable testing access.

ReNU syndrome is caused by variants in RNA gene RNU4-2 (RNA, U4 small nuclear 2), a non-coding RNA gene essential for the major spliceosome. This mechanism was described in detail by identifying pathogenic RNU4-2 variants in 73 unrelated individuals with NDD, establishing it as one of the most common non-coding contributors to these childhood conditions [1,2]. Both Chen et al. [1] and Greene et al. [2] demonstrate how highly recurrent variants in a constrained region disrupt spliceosomal function and underlie the condition. The RNU4-2 gene produces U4 snRNA, which also interacts with U6 snRNA to control activation of the spliceosome during pre-mRNA splicing [3]. This process is crucial for precise splice-site recognition and preventing premature spliceosome activity. Disease-causing variants in RNU4-2 are mainly found in highly conserved structural elements, such as stem III and the T-loop, which are essential for U4/U6 duplex stability and tri-snRNP assembly [4].

Genotype–phenotype correlations suggest that variant clustering within conserved structural domains of the U4 small nuclear RNA is associated with more severe neurodevelopmental outcomes [1]. These variants interfere with the proper splicing of introns, especially those crucial to the pediatric population’s healthy brain development and neurological function. Most individuals harbor the same recurrent single-nucleotide insertion as (n.64_65insT) in the RNU4-2 gene [1]. Clinically, symptoms like microcephaly, seizures, and speech delays are common to many other pediatric neurogenetic disorders, making underdiagnosis common. Reanalysis of undiagnosed neurodevelopmental cases identified recurrent RNU4-2 variants in 0.4% of patients, due to limited screening and awareness [2]. A 2.6% diagnostic yield was reported in cases unresolved by standard approaches [5]. In one case, a child with severe developmental delay went undiagnosed for years until a genome reanalysis finally revealed an RNU4-2 variant. Because standard tests often miss non-coding variants, families are left in long, frustrating diagnostic journeys [4].

ReNU syndrome is characterized by a distinctive pattern of dysmorphic facial features (e.g., hooded eyelids, full cheeks, tented philtrum, everted lower lip, and epicanthal folds) along with global developmental delay, hypotonia, and absent or limited speech, which have been described in multiple cohorts. Enhanced clinical recognition of this gestalt may facilitate earlier diagnosis and help prioritize individuals for appropriate genetic testing, particularly in settings lacking ready access to genomic sequencing. While other neurodevelopmental disorders such as Pitt-Hopkins, Rett, or Angelman-like conditions may share some clinical features (e.g., intellectual disability, limited speech), the distinctive facial pattern of ReNU syndrome can serve as a useful clue for differential diagnosis and early clinical suspicion [6].

Saturation genome editing has been used to test nearly all single-nucleotide variants in RNU4-2, creating a functional map that distinguishes disease-causing variants from benign ones. The study revealed a distinct recessive NDD associated with a different variant pattern [7]. Disease-linked variants in the 5′ stem-loop region have also been identified, along with a recurring clinical phenotype including hypotonia, feeding difficulty, and speech delay in a pediatric cohort [5]. Recent research has shown that spliceosomal non-coding RNA genes beyond RNU4-2 can underlie NDD. In particular, recurrent de novo variants in the RNU2-2 gene, which encodes a U2 small nuclear RNA component of the major spliceosome, have been identified as the cause of a distinct severe neurodevelopmental syndrome characterized by intellectual disability, autistic behaviors, microcephaly, hypotonia, epilepsy, and hyperventilation. In a cohort of affected individuals with recurrent de novo variants in RNU2-2, approximately 25 cases were reported, with an estimated prevalence of RNU2-2-associated disease approaching ~20 % of that observed for RNU4-2-associated disorders [8]. These findings underscore the clinical importance of non-coding RNA genes in neurodevelopmental disorder diagnostics.

To close existing diagnostic gaps, RNU 4-2 should be formally included in pediatric and newborn screening (NBS) protocols at both the institutional and national levels. This is now feasible and justified; a comprehensive variant map now supports the reliable interpretation of uncertain variants [1], while similar non-coding RNAs such as RNU4ATAC in Roifman syndrome have already been adopted into diagnostics despite initial complexity [9]. Pilot studies have shown that DNA methylation-based testing on dried blood spots can accurately identify Prader-Willi syndrome, a disorder caused by loss of the SNORD116-containing imprinted region on chromosome 15, demonstrating that methylation assays and related non-coding loci can be assessed early in life and suggesting the feasibility of integrating epigenetic markers into NBS workflows once validated and cost-effective methods are established [10].

NBS programs are traditionally designed to identify conditions in asymptomatic infants for which early intervention can prevent serious morbidity or mortality and for which reliable, cost-effective tests are available. These programs typically rely on biochemical assays that detect metabolic or hormonal abnormalities (e.g., phenylketonuria, congenital hypothyroidism) and are guided by established public health criteria such as the Wilson–Jungner principles and their modern adaptations [11]. Although genome-based approaches such as whole-genome sequencing (WGS) have the potential to detect rare genetic conditions that are not identified by standard biochemical NBS, evidence supporting the clinical utility, cost-effectiveness, and feasibility of population-wide genomic screening is currently limited. There are ongoing debates regarding the interpretation of genetic variants, the psychological impact of uncertain findings on families, consent processes, and the use of sequencing data beyond immediate clinical benefit [12].

At present, there are no gene-specific therapies for ReNU syndrome or many other NDD identified through genomic variation alone. For this reason, broad inclusion of genes such as RNU4-2 in universal NBS panels is premature under existing public health criteria, which emphasize actionable conditions with clear benefits to the newborn with available and effective early interventions [11]. Geographical and resource differences exacerbate diagnostic imbalances. Access to whole genome sequencing and advanced variant annotation systems is still limited in many low- and middle-income countries, where extended diagnostic journeys inflict a major emotional and financial strain on families. Children in these settings face prolonged diagnostic delays and inconsistent care. Underdiagnosis is costly for healthcare systems, necessitating multiple hospital visits, costly tests, and emergency care. We recommend that spliceosomal non-coding RNA genes including RNU4-2, RNU2-2, and others be systematically examined within diagnostic genomic sequencing workflows for infants and children presenting with unexplained developmental delay or NDD after initial first-tier evaluations. This targeted approach respects current ethical and policy frameworks while acknowledging the emerging role of genomic data in reducing diagnostic odysseys and informing supportive care and genetic counseling [12].

ReNU syndrome also highlights ethical issues in genomic medicine. Accurate interpretation in diverse populations is limited by the current variant databases’ bias towards European ancestry. Dominant variants in genes like RNU4-2 are often under-diagnosed or misclassified in non-European populations, escalating existing health inequalities [8]. This issue is even more pressing in low- and middle-income countries. This lack of diversity restricts accurate variant interpretation, and it becomes harder to link genetic variants to clinical findings in children from understudied backgrounds, leading to many unresolved or undiagnosed cases. Some clinical sequencing cohorts show that ancestry of the affected individuals affects determining how useful genetic results are, while some populations get clear diagnosis and others don’t, reinforcing existing health disparities [13]. At a global level, these biases raise ethical concerns, if genetic testing expands without inclusive data and fixing these biases, inequities in diagnosis and care may increase. Therefore, keeping the above-mentioned problems in mind, future research should prioritize including data from diverse populations and genomic testing should be interpreted keeping ancestry-aware variants in mind.

**However, there are still major hurdles, such as the lack of tools to connect genetic findings with clinical outcomes, limited agnostic testing options, and minimal genomics infrastructure in underserved regions where most RNU 4-2-related NDD cases are likely to occur. Further research is needed to explain the role of non-coding variants like RNU4-2 in disease manifestation. Establishing centralized phenotype registries and issuing guidelines for its inclusion in NDD gene panels could significantly improve diagnostic accuracy and timing.**

In conclusion, ReNU syndrome remains widely underdiagnosed—mainly due to RNU 4-2 being left out of standard testing, low clinical awareness, and overlapping symptoms. With better sequencing tools and variant tracking, we now have the chance to diagnose earlier and intervene sooner. Including RNU 4-2 in newborn screening, boosting clinician training, and improving global data sharing can help close the gap in neurodevelopmental care.

## Abbreviations

NDD: = Neurodevelopmental disorders.
NBS: = Newborn screening
